# Targeting CD47-mediated cancer senescence, a novel strategy for cancer immunotherapy

**DOI:** 10.3389/fimmu.2026.1815122

**Published:** 2026-07-21

**Authors:** Bei Zhao, Chunlu Liao, Hailian Wang, Mingyi Chen, Jianing Yang

**Affiliations:** 1 Department of Dermatology, Sichuan Academy of Medical Science and Sichuan Provincial People’s Hospital, University of Electronic Science and Technology of China, Chengdu, China; 2 Institute of Organ Transplantation, Sichuan Academy of Medical Science and Sichuan Provincial People’s Hospital, University of Electronic Science and Technology of China, Chengdu, China; 3 Clinical Immunology Translational Medicine Key Laboratory of Sichuan Province, Sichuan Provincial People’s Hospital, University of Electronic Science and Technology of China, Chengdu, China

**Keywords:** cancer senescence, CD47, chemotherapy, radiotherapy, SIRPα

## Abstract

A transmembrane protein that is extensively expressed, CD47 has an important role in the senescence of cancer cells. Therapy-induced senescence (TIS) by chemotherapy and radiotherapy restrains tumor proliferation yet drives immune evasion and treatment resistance, where CD47 acts as a core regulatory molecule linking cellular senescence and tumor immune escape. In this mini-review, we summarize the mechanisms governing CD47 upregulation during TIS, ranging from DNA damage response signaling, metabolic reprogramming and epigenetic modulation to post-transcriptional RNA modification. After addressing how CD47 is upregulated in TIS, we summarized the function of CD47 in both the maintaining the senescent statue and preventing senescent escape. Three major pathways are addressed. TSP-1/CD47 axis functions as both senescence maintenance and preventing escape, while, p16/c-MYC/CD47 axis and CD47/QPCT axis are responsible for senescence maintenance. Moreover, we summarize the therapeutic strategies toward CD47 blockade as s senolytic way, pointing out a novel strategy for oncotherapy. Overall, CD47 is a pivotal molecular bridge between TIS and immune tolerance. Future research on the role CD47 of cancer senescence, especially after chemotherapy or radiotherapy, could provide novel insight for oncotherapy.

## 1 Introduction

Senescence is an irreversible cell cycle arrest that can be caused by a variety of stressors, including chemotherapy and oncogene activation (1, 2). While senescence can suppress tumors, it can also promote tumor growth by secreting pro-inflammatory cytokines and other substances known as the senescence-associated secretory phenotype (SASP) (1, 3, 4). In return, SASP can promote tumor growth along with therapy resistance (5, 6). However, senescence can also act as a tumor-suppressive mechanism by inhibiting the division of damaged cells (7). Understanding the factors that contribute to cancer senescence is essential for developing appropriate cancer treatments.

CD47, a widely expressed transmembrane protein, is now recognized as a key regulator of cancer biology, particularly in cancer senescence (8). This protein interacts with TSP-1 and SIRPα, affecting cellular processes like proliferation, apoptosis, and immune evasion (9–11). Overexpression of CD47 in cancer is associated with poor prognosis and contributes to immune evasion by transmitting a “do not eat me” signal to macrophages, limiting phagocytosis (12–14). This interaction is critical for keeping cancer cells senescent, especially during chemotherapy-induced senescence. For example, studies have found that the TSP-1-CD47 interaction is critical for avoiding senescence escape in colorectal cancer, breast cancer, and ovarian cancer after chemotherapy (15–17). This interaction triggers p53-mediated DNA damage responses, which result in the overexpression of p16 and the downregulation of c-MYC, thereby maintaining the senescent state (18–20). CD47 inhibition has been shown in preclinical investigations to increase macrophage phagocytosis of cancer cells and improve anti-tumor immune responses (21). It has been used in a variety of cancers, including hematologic malignancies and solid tumors, with favorable outcomes. As a result, targeting CD47 has shown promise for increasing macrophage-mediated clearance of senescent cancer cells and improving treatment results (8, 22).

## 2 The activation of CD47 during therapy-induced senescence

For the direct evidence on how CD47 is activated during therapy-induced senescence, there is an important report. Chemotherapy-induced senescent cells secrete thrombospondin-1 (TSP1) to suppress senescence escape, a process tightly linked to reduced CD47 expression. Mechanistically, sustained senescence relies on functional p21. Upon p21 downregulation, c-MYC levels rise and bind to the CD47 promoter to repress CD47 transcription. This generates CD47^low^ cell populations that regain proliferative capacity, enabling tumor cells to evade chemotherapy-induced senescence and increased expression of CD47 is accompanied with chemotherapy-induced senescence (23). In this article, the authors found that CD47 drives cellular senescence. The interaction between TSP1 and CD47 promotes senescence, accompanied by increased ROS production, p53 activation, p21 upregulation and Rb hypophosphorylation. Meanwhile, CD47 could also preserve the senescent phenotype post chemotherapy. TSP1-CD47 signaling blocks senescence escape. Reduced CD47 expression correlates with diminished p21 and elevated Ki67 levels, supporting its functional role in sustaining cellular senescence.

During chemotherapy and radiotherapy, CD47 is upregulated and maintained at high levels via multiple mechanisms covering from transcriptional activation to post-translational regulation. Meanwhile, upregulated CD47 has been documented in a variety of cells, including cancer cell (23), epithelial cell (24), endothelial cell (25), mesenchymal stem cells (26), and etc. Because there are multiple mechanisms about therapy-induced CD47 activation, and CD47 activation results in cellular senescence. We will address how therapy will induce CD47 activation, hoping that it will draw attention to researchers to explore the senescence after CD47 activation.

### 2.1 DNA damage response induced by chemotherapy and radiotherapy

#### 2.1.1 Chemotherapy

Chemotherapeutic agents, such as cisplatin, could induce CD47 overexpression by inducing the phosphorylation of ATM and subsequent activation and phosphorylation of p65. In human hepatocellular carcinoma HepG2 and colon cancer RKO cells, cisplatin treatment upregulates CD47 expression alongside increased p65 phosphorylation. This upregulation can be abrogated by NF-κB inhibitors (27). In detail, cisplatin could upregulate CD47 expression via ATM/NF-κB signaling pathway. Cisplatin induced DDR could activate the phosphorylation of ATM and thereby activate NF-κB (p65), to upregulate CD47. Loss of CD47 could increase the cisplatin induced DDR via the inhibition of DDR related genes as *ERCC1, FANCA, BRCA2*, and thereby improve the sensitivity to cisplatin treatment (27). Cisplatin has been documented to enhance the expressions of CD47 in lung cancer cells (28).

A study on epithelial ovarian cancer patients who underwent neoadjuvant chemotherapy revealed that the CD47 expression levels increased post therapy. And *in vitro* study using cisplatin to treat the ovarian cancer cell lines also suggested the increased expression of CD47 (29). Another study suggested that radiotherapy combined with glutamine inhibition could increase CD47 expression (30).

#### 2.1.2 Radiotherapy

Irradiation (IR) and other genotoxic therapy could induce DNA double-strand break (DSB). In response to DSB, γH2AX gathered at the break foci. Followed by the recruiting of MRN complex, composed by Mre11, Rad51 and Nbs1, at the DSB foci, the researchers found there were upregulation of CD47. Cells treated with Mre11 inhibitor fail to increase CD47 expression and do not respond to DDR (31). This suggest DSB recruited γH2AX-MRN complex lead to the activation of CD47.

In colorectal cancer, IR triggers the activation of the ATR-Chk1 signaling axis, which subsequently drives STAT3 phosphorylation to increase CD47 transcription and elevated post-IR CD47 expression levels in various human solid tumor cells. Treatment with either ATR or Chk1 inhibitors reduces the IR-mediated upregulation of both CD47 and PD-L1 (32).

### 2.2 Metabolic reprogram

Radiotherapy changes the tumor microenvironment (TME) and results in metabolic reprogram. In glioblastoma stem cells (GSCs), CD47 expression is elevated following radiotherapy, and this upregulation correlates with radio resistance. Meanwhile, radiation reshapes the TME and drives phenotypic switching in tumor cells. In detail, IR activates AMPK signaling to upregulate CD47 through two epigenetic mechanisms. The first one is that activated AMPK sustains hyperphosphorylation of the retinoblastoma protein RB to keep it dissociated from KDM5A. Blocking KDM5A-mediated demethylation and thus increasing H3K4 methylation at the CD47 promoter. The second one is IR also increases HDAC7-dependent H3K27 acetylation at the CD47 locus, jointly elevating CD47 to evade macrophage phagocytosis and stimulate macrophages toward an M2-like phenotype. As a contrast, radiosensitive non-stem glioma cells feature hypophosphorylated RB at S807/S811 that binds KDM5A and reduces CD47 promoter H3K4 methylation to limit CD47 expression (33).

Enhanced tumor immune evasion after chemotherapy also contribute to CD47 expression after therapy. In osteosarcoma tissues, chemotherapy markedly increases CD47 expression, which is positively correlated with patient mortality. Mechanistically, macrophage-secreted IL-18 promotes LAT2-mediated uptake of leucine and glutamine in tumor cells, subsequently activating mTORC1 and driving c-MYC-dependent CD47 transcription (34).

### 2.3 Signaling pathway

#### 2.3.1 DDR signaling

- MRE11/MRN complex dependency: CD47 upregulation induced by ionizing radiation or other genotoxic agents is correlated with DSBs. Loss of MRE11 function or treatment with the MRE11 inhibitor mirin abolishes CD47 induction upon DNA damage, identifying the MRN complex as a critical upstream sensor driving CD47 upregulation (31).
- ATR-Chk1-STAT3: In colorectal cancer, radiotherapy triggers ATR and Chk1 activation to stimulate STAT3 phosphorylation, which subsequently enhances CD47 transcription. Inhibitors targeting either ATR or Chk1 attenuate radiation-mediated upregulation of both CD47 and PD-L1 (32).
- ATM/NF-κB: In hepatocellular carcinoma cells, cisplatin upregulates CD47 through the ATM/NF-κB pathway. Either ATM inhibition or p65 knockdown abrogates cisplatin-induced CD47 elevation (27).

#### 2.3.2 Transcriptional regulation driven by metabolic reprogramming

- Fatty acid oxidation (FAO)-dependent NF-κB/RelA acetylation: In radioresistant glioblastoma, acetyl-CoA generated from fatty acid oxidation (FAO) promotes acetylation of NF-κB/RelA to stimulates CD47 transcription (35).
- mTORC1-c-Myc signaling cascade: In osteosarcoma, chemotherapy stimulates macrophages to secrete IL-18. This cytokine enhances LAT2-facilitated uptake of leucine and glutamine in tumor cells, leading to mTORC1 activation and subsequent c-Myc-dependent CD47 transcription (34).

#### 2.3.3 Epigenetic transcriptional regulation

AMPK-mediated H3K4 trimethylation and H3K27 acetylation: In radioresistant glioblastoma stem cells, irradiation suppresses KDM5A expression in an AMPK-dependent manner, which elevates H3K4 methylation at the CD47 promoter. Concurrently, HDAC7 increases H3K27 acetylation at the same promoter locus, and these two epigenetic modifications synergistically activate CD47 transcription (33).

#### 2.3.4 Post-translation regulation

M^6^A RNA methylation: In EGFR-TKI-resistant NSCLC, downregulated ALKBH5 leads to increased m^6^A modification and improved stability of CD47 mRNA, ultimately increasing CD47 protein abundance (36).

## 3 CD47 contributes to the maintenance of senescence

### 3.1 TSP-1/CD47 axis, a cell-intrinsic pathway involved in senescence maintenance and prevent senescent escape

CD47, a widely expressed transmembrane protein, regulates cellular processes through interactions with TSP-1 and SIRPα. The TSP-1-CD47 axis controls a variety of biological activities, including cell proliferation, apoptosis, and immunological regulation (17, 37). TSP-1, a matricellular protein, attaches to CD47 to regulate cell activities, which can promote or inhibit cell proliferation depending on the cellular context (38). The TSP-1-CD47 interaction has been found to influence cancer progression and senescence (38). For example, TSP-1 has been linked to the production of cellular senescence in endothelial cells and cancer cells, which can have both tumor-suppressive and tumor-promoting effects (38). This dual role highlights the complexities of the TSP-1-CD47 axis in cancer biology (39).

The TSP-1/CD47 signaling pathway specifically promotes and stably maintains cellular senescence phenotypes through a typical cell-intrinsic pathway, which is distinct from the extrinsic senescence regulation mediated by the external microenvironment. As an upstream trigger signal, the secreted matricellular protein TSP-1 specifically binds to the cell membrane surface receptor CD47 and initiates intracellular autonomous signal transduction cascades, serving as a core trigger for activating cell-intrinsic senescence. It has been documented that the binding of TSP-1 to CD47 further activates NOX1, induces massive intracellular reactive oxygen species (ROS) production, and subsequently triggers the p53-mediated DNA damage response pathway (38). This process upregulates the cyclin-dependent inhibitor p21 and inhibits Rb protein phosphorylation, ultimately leading to persistent cell cycle arrest and driving cells into a senescent state (38). Accumulating studies in cellular and tissue models have validated the pivotal role and tissue-specific features of the TSP-1/CD47 signaling axis in senescence maintenance. In endothelial cell senescence models, TSP-1/CD47 signaling acts as a core mechanism driving endothelial replicative senescence. CD47 deficiency significantly improves angiogenic function and alleviates replicative senescence, whereas TSP-1 accelerates endothelial cell cycle arrest and senescence progression in a CD47-dependent manner (25).

Notably, this signaling axis functions not only in senescence induction, but also in prevention of senescence escape. A specific feedback loop formed by CD47, p21, and c-MYC is critical for its senescence-maintaining function. In another study, TSP-1 is generated by senescent cell and its function is to prevent senescent escape by increasing the expression of CD47 via the p21^Waf1^/c-Myc axis. p21 inactivation markedly upregulates c-MYC expression, and aberrantly activated c-MYC in turn modulates CD47 expression levels. This bidirectional molecular regulatory network locks cellular senescence phenotypes, effectively inhibiting senescence escape of tumor cells after chemotherapy and stabilizing the senescent state (23).

Furthermore, TSP-1-CD47 interaction is very important in the onset and maintenance of senescence in cancer cells. This interaction can trigger p53-mediated DNA damage responses, causing cell cycle arrest and the onset of a senescent state (8). For example, investigations have demonstrated that TSP-1-induced senescence in endothelial cells is dependent on its binding to CD47 (8). TSP-1 loss resulted in lower survival and enhanced cancer aggression in a mouse model of Kras-induced lung carcinogenesis, underscoring TSP-1’s role in preserving oncogenic Ras activation-induced senescence (40, 41). Furthermore, the TSP-1-CD47 axis has been demonstrated to cause senescence in human pulmonary artery endothelial cells at levels seen in diseased patients’ plasma (38, 42). This implies that the TSP-1-CD47 signaling pathway promotes cellular senescence in endothelial cells, which could have significance for senotherapy in vascular disorders (23, 43).

### 3.2 p16/c-MYC/CD47 axis and CD47/QPCT axis for senescence maintenance

P16 could also promote CD47 expression to maintain senescence (44). When p16 is downregulated in senescent cells, c-MYC expression rises. c-MYC then binds to the CD47 promoter and represses transcription, leading to the formation of CD47-low cells that escape senescence (45). The p16-c-MYC-CD47 axis leads to senescence maintenance and prevent senescent escape in cancer cells (44). Specifically, activation of p16 can result in downregulation of c-MYC (46), and it increases CD47 levels according to previous findings (44). This ultimately leads to telomere-independent senescence. The reciprocal interaction between p16, c-MYC, and CD47 is critical for maintaining the senescent state. When p16 is activated, c-MYC expression is inhibited, which can enhance CD47 levels and potentially induce senescence state. Furthermore, upregulation of the CD47-QPCT/L axis contributes to the senescent cells inhibiting the clearance by macrophages, which means that when CD47 is upregulated, there will be more senescent cells (24). These data suggest that targeting CD47 contribute to the senescence maintenance via p16/c-Myc and CD47-QPCT/L axis.

## 4 CD47/SIRPα signaling: an immune-evasion mechanism that protects senescent cells from phagocytosis

CD47 enables senescent cancer cells to resist immune clearance, resulting in sending a “Don’t eat me” signal to macrophages, and preventing their clearance. The interaction of CD47 and SIRPα on macrophages limits their phagocytic activity, allowing cancer cells to avoid immune monitoring (23). CD47, a marker that inhibits macrophage phagocytosis by attaching to SIRPα on phagocytes. Recent research shows that senescent cells upregulate CD47 in comparison to normal proliferating cells, which inhibits macrophage-mediated clearance (23). This overexpression causes senescent cells to concentrate in tissues rather than being eliminated by the immune system. Targeting CD47 with antibodies or other treatments can disrupt this connection, allowing macrophages to phagocytose senescent cancer cells more efficiently (47). *In vitro* and *in vivo*, senescent models overexpress CD47, which prevents macrophages from recognizing them (48). Clinical trials show that inhibiting the SIRPa-CD47-SHP-1 axis with a CD47 antagonist eliminates SnCs and maintains tissue homeostasis (49). This form of therapy is frequently applied in cancer research and could be explored as a senolytic treatment for chronic disorders (50). Initially reported in 1996, 4N1K (a TSP-1 mimic peptide and a potential agonist of CD47), can greatly increase cell spreading on porphyrin by binding to IAP (without affecting adhesion), which used C32 human melanoma cells expressing IAP, αvβ3, and αvβ5 (51). But later, studies revealed the activity of 4N1K is independent on CD47 (52–55). Although there is a study using 4N1Ks as a senolytic treatment in the regulation of CD47 pathway in senescence (56), but this study fails to be more convincing due to lacking of appropriate controls. Interestingly, CD47 expression differs depending on how senescence is triggered. In chemotherapy-induced senescence, CD47 is initially maintained but can be downregulated during senescence escape (23, 29). Furthermore, cytokine-induced senescence (CIS) reduces CD47 expression in senescent cancer cells, potentially increasing their susceptibility to immune detection (57). Senescent cell surveillance is suppressed by inhibitory immunological checkpoints, which encourage the accumulation of these cells when ageing or ageing-related diseases (58).

Moreover, TSP1/CD47 signaling in T and NK cells also plays important roles and can be targeted therapeutically. In the TME, CD47 signaling can reduce the synergistic effect of radiotherapy and T cell immunity, neutralize the anti-tumor immune response elicited by radiation, and suppress the activation, infiltration, and cytotoxic function of antigen-specific T cells (59). According to a different study, CD47 does not significantly affect T cell development, but it can control CD8^+^ T cell function in a context-dependent way. Under short-term stimulation, CD47 deficiency can improve the effector phenotype and proliferative capacity of CD8^+^ T cells, whereas prolonged T cell receptor stimulation will inhibit their effector function and promote apoptosis (60). By blocking the hydrogen sulfide signaling pathway and the production of associated enzymes, TSP1/CD47 signaling can further prevent T cell activation (61). TSP1 binding to CD47 reduces T cell glycolysis, promotes metabolic reprogramming, and hinders the anti-tumor activity of CD8^+^ T cells, according to another study (62). TSP1/CD47 signaling can cause CD8^+^ T cell malfunction, activate the calcineurin-NFAT-TOX signaling pathway, increase the expression of inhibitory receptors like PD-1 and LAG-3, and decrease the release of effector molecules like TNF and IFN-γ. Targeting CD47 can reverse the inhibitory effects, restore T cell effector function, and metabolic levels, making it a promising target for improving anti-tumor immune responses (39).

## 5 Therapeutic CD47 blockade as a senolytic-like or immunotherapeutic strategy

### 5.1 Combination therapies

Combining CD47 inhibitors with chemotherapy is an intriguing approach for increasing the efficacy of cancer treatments. Chemotherapy can cause senescence in cancer cells, leaving them more vulnerable to immune-mediated clearance. However, these senescent cells frequently avoid immune monitoring by upregulating CD47, which sends a “do not eat me” signal to macrophages. By suppressing CD47, we can disrupt this protective signal, allowing macrophages to more efficiently phagocytose senescent cancer cells (57, 63). For example, studies have found that combining CD47 inhibitors with chemotherapeutic medicines such as anthracyclines can considerably improve tumor clearance in triple-negative breast cancer models (64). BCL-2 inhibitors and anti-CD47 immunotherapy collaborate to improve anti-tumor activity in B-cell lymphoma (65). This combination reduces tumor burden while also preventing senescent cells from escaping, overcoming treatment resistance. However, this technique presents multiple challenges, such as regulating elevated immunological activity, which may result in immune-related adverse events (irAEs). To balance treatment benefits against potential adverse effects, novel neutralizing antibody for CD47 has been generated to reduce the irAEs (66).

### 5.2 Synergistic effects with other immunotherapies and targeted therapies

The combination of CD47 inhibitors with other immunotherapies and targeted treatments has demonstrated substantial promise for improving anti-tumor activity (67). Immune checkpoint inhibitors (ICIs), such as PD-1/PD-L1 inhibitors, have been shown to work synergistically with CD47 inhibitors. For example, in preclinical models of non-small cell lung cancer (NSCLC), combining CD47 inhibitors with PD-1 inhibitors improved T-cell activation and tumor control (68). Similarly, targeted therapy like as PARP inhibitors and histone deacetylase (HDAC) inhibitors, has been tested in conjunction with CD47 inhibitors (69). HDAC inhibitors have shown potential in improving the efficacy of CD47-targeted therapies by modifying the TME and increasing macrophage-mediated phagocytosis (70). For example, in melanoma models, the combination of HDAC6 inhibitors with CD47-blocking antibodies has been found to drastically restrict tumor development by increasing macrophage phagocytic activity (71).

### 5.3 Clinical trials

The clinical development of CD47-targeted medicines has increased significantly in recent years (72). Clinicaltrials.gov currently lists several trials targeting CD47 (**Table 1**), anti-CD47 bispecific antibody (**Table 2**), trials targeting SIRPα-Fc fusion proteins (**Table 3**), and trials targeting SIRPα (**Table 4**). These studies involve a variety of cancer types, including hematologic malignancies, solid tumors, and ovarian cancer. CD47 inhibitors have shown some promise in treating hematologic malignancies such as acute myeloid leukemia (AML) and non-Hodgkin lymphoma (NHL) (73, 74). For example, the monoclonal antibody magrolimab (Hu5F9-G4) has shown some efficacy in AML patients when combined with azacitidine, with complete remission (CR) rates reaching up to 32.2% in some cohorts (75). Similarly, in relapsed/refractory indolent NHL, magrolimab coupled with rituximab has demonstrated objective response rates (ORR) was 52.2%, with 30.4% achieving a complete response (76). However, recent development of magrolimab has faced major setbacks. Gilead discontinued the ENHANCE-3 phase III AML study due to futility and reported a full FDA clinical hold for magrolimab studies in AML/MDS (https://www.gilead.com/company/company-statements/2024/gilead-statement-on-discontinuation-of-phase-3-enhance-3-study-in-aml). Subsequent ENHANCE-3 data showed that adding magrolimab to venetoclax and azacitidine did not improve overall survival. A total of 378 patients were enrolled in ENHANCE-3 (NCT05079230). It is a phase 3 double-blind placebo-controlled trial for AML patients unfit for intensive chemotherapy to test magrolimab combined with venetoclax and azacitidine. The trial was terminated due to futility at interim analysis. Compared with the venetoclax–azacitidine control arm (median OS 14.1 months, 6-cycle CR rate 46.0%), the magrolimab combination showed inferior survival (median OS 10.7 months, HR = 1.178) and a lower CR rate (41.3%), alongside more fatal adverse events (19.0% *vs* 11.4%), mostly severe grade 5 infections and respiratory complications. While common hematologic and infectious toxicities were balanced between arms, magrolimab brought extra lethal risks. Consistent with the futility outcome of the parallel ENHANCE-2 trial focusing on TP53-mutant AML, this study demonstrates that adding magrolimab to standard low-intensity doublet therapy offers no clinical benefit and worsens safety, highlighting major challenges for anti-CD47 antibody development in AML treatment (77).

**Table 1:** Clinical trials for oncotherapy with an anti-CD47 monoclonal antibody.

NCT number	Drug name (alternative name)	Type of tumor	Target	Status	Phase
NCT02953509	Magrolimab (Hu5F9-G4)	Relapsed/Refractory B-cell Non-Hodgkin's Lymphoma	CD47	Terminated	Phase 1b/2
NCT05738161	Magrolimab (Hu5F9-G4)	Advanced Urothelial Carcinoma	CD47	Withdrawn	Phase 2
NCT05835011	Decitabine/Cedazuridine + Magrolimab (Hu5F9-G4)	Intermediate- to Very High-Risk Myelodysplastic Syndromes (MDS)	CD47, DNA methyltransferase	Terminated	Phase 2
NCT03248479	Magrolimab (Hu5F9-G4)	Hematological Malignancies (AML/MDS/Lymphoma)	CD47	Terminated	Phase 1b/2
NCT05829434	Magrolimab (Hu5F9-G4)	Newly Diagnosed AML, HR-MDS	CD47	Withdrawn	Phase 3
NCT03869190	Atezolizumab ± Multiple Immunotherapy Combinations (MORPHEUS-UC)	Bladder Cancer, Urothelial Carcinoma	PD-L1	Completed	Phase 1b/2
NCT04313881	Magrolimab (Hu5F9-G4) + Azacitidine	Untreated Myelodysplastic Syndrome (MDS)	CD47, DNA methyltransferase	Terminated	Phase 3
NCT04435691	Magrolimab (Hu5F9-G4) + Azacitidine + Venetoclax	Recurrent/Refractory Acute Myeloid Leukemia (AML)	CD47, DNA methyltransferase, BCL-2	Terminated	Phase 1b/2
NCT05823480	Magrolimab (Hu5F9-G4) + Azacitidine	High-Risk AML/MDS Post Allogeneic HCT	CD47, DNA methyltransferase	Withdrawn	Phase 2
NCT02216409	Magrolimab (Hu5F9-G4)	Advanced Solid Tumors	CD47	Completed	Phase 1
NCT02678338	Magrolimab (Hu5F9-G4) (CAMELLIA)	AML, MDS	CD47	Completed	Phase 1b/2
NCT03922477	Atezolizumab + Magrolimab (Hu5F9-G4)	Relapsed/Refractory AML	CD47, PD-L1	Terminated	Phase 1b
NCT04751383	Magrolimab (Hu5F9-G4) + Dinutuximab	Relapsed/Refractory Neuroblastoma, Recurrent Osteosarcoma	CD47, GD2	Completed	Phase 1/2
NCT04541017	Magrolimab (Hu5F9-G4) + Mogamulizumab	Cutaneous T-Cell Lymphoma (Mycosis Fungoides/Sezary Syndrome)	CD47, CCR4	Terminated	Phase 2
NCT05807126	Magrolimab (Hu5F9-G4) + Olaparib	BRCA-mutated Metastatic Breast Cancer, Castrate-Resistant Prostate Cancer	CD47, PARP	Withdrawn	Phase 2
NCT04788043	Magrolimab (Hu5F9-G4) + Pembrolizumab	Relapsed/Refractory Classic Hodgkin Lymphoma	CD47, PD-1	Active, not recruiting	Phase 2
NCT03527147	Multi-agent Immunotherapy Platform (PRISM)	Relapsed/Refractory Aggressive B-NHL (DLBCL)	Multiple immune targets	Completed	Phase 1b/2
NCT02953782	Magrolimab (Hu5F9-G4) + Cetuximab	Solid Tumors, Advanced Colorectal Cancer	CD47, EGFR	Completed	Phase 1b/2
NCT03558139	Magrolimab (Hu5F9-G4) + Avelumab	Platinum-refractory Ovarian Cancer, Solid Tumors	CD47, PD-L1	Completed	Phase 1b/2
NCT03717103	IBI188	Advanced Malignancies (Solid/Hematologic)	CD47	Completed	Phase 1
NCT06789848	Ligufalimab (AK117) + Cadonilimab	Advanced Hepatocellular Carcinoma, Biliary Tract Cancer	CD47, PD-L1/CTLA-4	Recruiting	Phase 1b/2
NCT07264075	Ivonescimab ± Ligufalimab (AK117)	Squamous Cell Carcinoma of Head and Neck (SCCHN)	PD-1/VEGF, CD47	Recruiting	Phase 2
NCT03834948	AO-176	Advanced Solid Tumors	CD47	Completed	Phase 1
NCT04445701	AO-176 ± Bortezomib/Dexamethasone	Relapsed/Refractory Multiple Myeloma	CD47, Proteasome	Completed	Phase 1b/2
NCT04257617	ZL-1201	Advanced Malignancies (Solid/Hematologic)	CD47	Completed	Phase 1
NCT03512340	SRF231	Advanced Solid & Hematologic Cancers	CD47/SIRPα	Completed	Phase 1
NCT05266274	Unspecified Anti-CD47 Monoclonal Antibody + Azacitidine	Post-transplant Recurrent AML	CD47, DNA methyltransferase	Unknown status	Phase 2

**Table 2:** Clinical trials for oncotherapy with an anti-CD47 bispecific antibody.

NCT number	Drug name (alternative name)	Type of tumor	Target enrollment	Status	Phase
NCT04328831	IBI322 (CD47/PD-L1)	Advanced Malignancies	218	Recruiting	Phase 1
NCT04338659	IBI322 (CD47/PD-L1)	Advanced Malignancies	45	Recruiting	Phase 1
NCT04795128	IBI322 (CD47/PD-L1)	Hematological Malignancies	230	Recruiting	Phase 1
NCT04912466	IBI322 (CD47/PD-L1)	Solid Tumor	36	Recruiting	Phase 1
NCT04097769	HX009 (CD47/PD-1)	Advanced Solid Tumor	20	Active, not recruiting	Phase 1
NCT04886271	HX009 (CD47/PD-1)	Solid Tumor	210	Recruiting	Phase 2
NCT05189093	HX009 (CD47/PD-1)	Lymphoma	99	Recruiting	Phase 2
NCT03804996	TG-1801 (NI-1701, CD47/CD19)	B-Cell Lymphoma	50	Active, not recruiting	Phase 1
NCT04806035	TG-1801 (NI-1701, CD47/CD19)	B-Cell Lymphoma, CLL	60	Recruiting	Phase 1
NCT04746131	IMM0306 (CD47/CD20)	CD20-positive B-cell NHL	90	Not yet recruiting	Phase 1
NCT05805943	IMM0306 (CD47/CD20)	B-NHL	–	Recruiting	Phase 1/2
NCT05771883	IMM0306 (CD47/CD20) + Lenalidomide	B-NHL	–	Recruiting	Phase 1/2
NCT04853329	JMT-601 (CPO-107, CD47/CD20)	CD20-positive B-cell NHL	–	Recruiting	Phase 2
NCT05403554	NI-1801 (CD47/MSLN)	Epithelial Ovarian Cancer, TNBC, NSCLC	40	Recruiting	Phase 1
NCT04881045	PF-07257876 (CD47/PD-L1)	Ovarian Cancer, NSCLC	28	Active, not recruiting	Phase 1
NCT05780307	IMM2520 (CD47/PD-L1)	Advanced Solid Tumor, NSCLC, Breast Cancer, HNSCC, CRC	48	Recruiting	Phase 1
NCT04980690	IBC0966 (CD47/PD-L1)	Advanced Malignant Tumors including TNBC	228	Recruiting	Phase 1/2
NCT04328831	IBI322 (CD47/PD-L1)	Advanced Malignancies	218	Recruiting	Phase 1
NCT04338659	IBI322 (CD47/PD-L1)	Advanced Malignancies	45	Recruiting	Phase 1
NCT04795128	IBI322 (CD47/PD-L1)	Hematological Malignancies	230	Recruiting	Phase 1
NCT04912466	IBI322 (CD47/PD-L1)	Solid Tumor	36	Recruiting	Phase 1
NCT04097769	HX009 (CD47/PD-1)	Advanced Solid Tumor	20	Active, not recruiting	Phase 1
NCT04886271	HX009 (CD47/PD-1)	Solid Tumor	210	Recruiting	Phase 2
NCT05189093	HX009 (CD47/PD-1)	Lymphoma	99	Recruiting	Phase 2
NCT03804996	TG-1801 (NI-1701, CD47/CD19)	B-Cell Lymphoma	50	Active, not recruiting	Phase 1

**Table 3:** Clinical trials for oncotherapy with SIRPα-Fc fusion proteins.

NCT number	Drug name (alternative name)	Type of tumor	Target enrollment	Status	Phase
NCT03013218	Evorpacept (ALX148)	Metastatic Cancer, Solid Tumor, NHL	174	Active, not recruiting	Phase 1
NCT04417517	Evorpacept (ALX148)	Higher Risk MDS	65	Active, not recruiting	Phase 1/2
NCT04755244	Evorpacept (ALX148)	AML	97	Active, not recruiting	Phase 1/2
NCT05025800	Evorpacept (ALX148)	B-Cell NHL	47	Recruiting	Phase 1/2
NCT04675294	Evorpacept (ALX148)	Head and Neck Squamous Cell Carcinoma	111	Recruiting	Phase 2
NCT05002127	Evorpacept (ALX148)	Gastric/GEJ Cancer	450	Not yet recruiting	Phase 2/3
NCT02663518	TTI-621	Hematological Malignancies, Solid Tumor	-	Terminated	Phase 1
NCT02890368	TTI-621	Solid Tumors, Mycosis Fungoides	-	Terminated	Phase 1
NCT03530683	TTI-622	Lymphoma, MM, AML, DLBCL	177	Active, not recruiting	Phase 1
NCT05139225	TTI-622	Multiple Myeloma	32	Recruiting	Phase 1
NCT05675449	TTI-622	Multiple Myeloma	14	Recruiting	Phase 1
NCT05833984	IMM-01	Hodgkin Lymphoma	309	Recruiting	Phase 1/2
NCT05140811	IMM-01	MDS/AML	-	Recruiting	Phase 1/2

**Table 4:** Clinical trials for oncotherapy with an anti-SIRPα antibody.

NCT number	Drug name (alternative name)	Type of tumor	Status	Phase
NCT03990233	BI 765063 (Anti-SIRPα)	Advanced Solid Tumors	Recruiting	Phase 1
NCT04653142	BI 765063 (Anti-SIRPα)	Ovarian Cancer	Recruiting	Phase 1
NCT05249426	BI 765063 (Anti-SIRPα)	Solid Tumors	Recruiting	Phase 1
NCT05446129	BI 765063 (Anti-SIRPα)	Solid Tumors	Recruiting	Phase 1
NCT05068102	BI 765063 (Anti-SIRPα)	Solid Tumors	Recruiting	Phase 1
NCT05765851	DS-1103a (Anti-SIRPα)	Breast Cancer, Advanced Solid Tumor	Recruiting (78)	Phase 1
NCT04406623	SL-172154 (SIRPα-Fc-CD40L)	Platinum-Resistant Ovarian Cancer	Recruiting	Phase 1
NCT03990233	BI 765063 (Anti-SIRPα)	Advanced Solid Tumors	Recruiting	Phase 1
NCT04653142	BI 765063 (Anti-SIRPα)	Ovarian Cancer	Recruiting	Phase 1
NCT05249426	BI 765063 (Anti-SIRPα)	Solid Tumors	Recruiting	Phase 1
NCT05446129	BI 765063 (Anti-SIRPα)	Solid Tumors	Recruiting	Phase 1
NCT05068102	BI 765063 (Anti-SIRPα)	Solid Tumors	Recruiting	Phase 1
NCT04406623	SL-172154 (SIRPα-Fc-CD40L)	Platinum-Resistant Ovarian Cancer	Recruiting	Phase 1
NCT03990233	BI 765063 (Anti-SIRPα)	Advanced Solid Tumors	Recruiting	Phase 1

In solid tumors, the combination of CD47 inhibitors with various therapies is being investigated. Patients with platinum-resistant ovarian cancer participated in the first-in-human phase I trial of SL-172154, a bispecific CD47 inhibitor and CD40 agonist Fc-fusion protein, and they demonstrated promising efficacies (78). Furthermore, in breast cancer, combinations of CD47 inhibitors with HER2-targeted treatments such as trastuzumab have shown some decreases in tumor growth and better therapeutic success (79).

## 6 Challenges and perspectives

Future studies on CD47 in cancer senescence should concentrate on a few crucial areas. First, more research is needed into the mechanisms of CD47 in various cancer types. While CD47’s role in immune evasion is well understood, its contribution to senescence maintenance and escape pathways needs to be further explored. For example, CD47 inhibition has demonstrated some success in hematologic cancers, while the outcomes in solid tumors have been less consistent. This mismatch indicates that the TME and the unique cellular environment alter CD47 function, needing a better knowledge of these interactions. Second, the identification of predictive biomarkers is critical for selecting patients who will benefit most from CD47-targeted treatments. This will allow for more tailored treatment approaches and better therapeutic outcomes. Furthermore, investigating the efficacy of CD47 inhibitors in combination with other drugs, including chemotherapy, immunotherapies, and targeted therapies, is a promising direction. In preclinical models, combining CD47 inhibitors with additional medicines improves antitumor activity and overcomes resistance mechanisms. Furthermore, discovering and overcoming resistance mechanisms to CD47 inhibitors will be critical for long-term success. This includes investigating the role of the TME and devising ways to increase immune cell infiltration and activation. Finally, we still need to investigate novel CD47 targets and mechanisms in cancer senescence. Investigating the connections between CD47 and other signaling pathways, such as TSP-1 and p53, will lead to new treatment targets and combination therapies.

However, the application of CD47 inhibitors in combination therapies must be carefully considered to prevent stimulating senescence escape and chemotherapy resistance. While combination medicines show great promise, they also offer risks such as increased toxicity and the potential to promote resistance. For example, excessive doses of CD47 inhibitors might cause serious side effects such as hemolysis and thrombocytopenia. Thus, adjusting dose regimens and properly monitoring patients for side effects are critical. Furthermore, the TME determines the efficacy of CD47 inhibitors. In some situations, combining CD47 inhibitors with anti-angiogenic treatments has improved therapeutic outcomes by creating a more favorable immunological environment. This emphasizes the significance of understanding the interactions between CD47 and other signaling pathways within the TME.

## 7 Conclusion

In conclusion, CD47 has been documented to be overexpressed after chemotherapy or radiotherapy, and the elevated CD47 is correlated with senescence in a broad range of cell types. There is also a report about CD47 mediated senescence after therapy. Future research should focus on the understanding of the processes of CD47 in TIS, including how CD47 is upregulated in TIS and how it maintained the senescent phenotype for cancer cells to escape elimination. Strategies toward CD47 mediated TIS may provide a novel therapeutic way for oncotherapy.
